# Relationship between walking distance within the first 24 h following lung cancer surgery and clinical outcomes

**DOI:** 10.1007/s11748-025-02139-w

**Published:** 2025-03-24

**Authors:** Makoto Asaeda, Yukio Mikami, Akihiro Matsumoto, Yuki Nakashima, Kouki Fukuhara, Tomoya Hirai, Atsushi Kamigaichi, Norifumi Tsubokawa, Takahiro Mimae, Yoshihiro Miyata, Morihito Okada

**Affiliations:** 1 Division of Rehabilitation, Department of Clinical Practice and Support, Hiroshima University Hospital, 1-2-3, Kasumi, Minami-ku, Hiroshima, 734-8551 Japan; 2 Department of Rehabilitation Medicine, Hiroshima University Hospital, 1-2-3 Kasumi, Hiroshima, 734-8551 Japan; 3 Department of Surgical Oncology, Hiroshima University, Hiroshima, 734-8551 Japan

**Keywords:** Lung neoplasms, Rehabilitation, Exercise therapy, Walk test, Length of stay

## Abstract

**Objectives:**

Lung cancer remains a major health concern in Japan, with over 126,000 cases diagnosed in 2019. Surgery is the primary treatment for stage I–III non-small-cell lung cancer. The 6-min walk test is widely used to assess physical endurance before and after surgery, with preoperative distances below 500 m associated with prolong hospital stays. Postoperatively, endurance typically decreases by 50–100 m. Early mobilization is critical to prevent this decline; however, no clear consensus exists on optimal rehabilitation protocols after lung cancer surgery.

**Methods:**

This retrospective cohort study examined the relationship between early postoperative walking distance and clinical outcomes in 104 patients who underwent lung cancer surgery between 2020 and 2023. Physical function was assessed using the 6-min Walk Test before admission and before discharge.

**Results:**

A significant correlation was found between the distance walked within the first 24 h after surgery and the pre- and postoperative 6-min walk test performance. However, no significant association was observed between early walking distance and length of hospital stay or postoperative complications.

**Conclusions:**

Early mobilization after lung cancer surgery aligns closely with preoperative endurance levels, suggesting that improving preoperative physical function can enhance postoperative recovery and reduce complications. Further research is needed to standardize the rehabilitation protocols.

## Introduction

Lung cancer remains a major health concern in Japan, with 126,548 cases diagnosed in 2019 and rising incidence and mortality rates [1]. For non-small cell lung cancer (NSCLC) at clinical stages I to III, surgical treatment is recommended when deemed appropriate, particularly up to clinical stage IIa. A meta-analysis of clinical outcomes following lung cancer surgery demonstrated that surgical treatment for stage I–III NSCLC demonstrates better outcomes than conservative treatment [2]

The 6-min Walk Test (6MWT) for physical function is a common measure of overall endurance, regardless of lung cancer surgery [3]. Preoperative distances typically ranges from 450 to 500 m, with values below 500 m linked to longer hospital stays [4, 5]. Postoperative endurance, as assessed by the 6MWT, decreases by 50–100 m on average, even in the absence of complications [4, 6]. Postoperative rehabilitation, including exercise training, can improve endurance, with reported gain of 62.83 m over 12 months [7]. The walking distance in the 6MWT before and after surgery is widely used as an outcome measure for lung cancer treatment.

Traditional postoperative care following thoracic surgery often prolong bed rest in the first few hours or days [8]. However, postoperative immobility is a major risk factor for deviation from enhanced recovery after surgery (ERAS) protocols and prolonged length of stay (LOS) following colorectal surgery [9]. Additionally, immobility is associated with increased morbidity and LOS following lung cancer resection [10]. A study on early postoperative walking observed that patients walked for 8.26 min (54.94 m) after 34.18 h postoperatively [11]. Implementing a protocol for mobilization within 4 h after surgery could shorten the drainage period [12]; however, it could not reduce the rate of minor complications [13]. Moreover, consensus on perioperative rehabilitation treatment is lacking [14, 15], and systematic reviews have not demonstrated the benefits of early mobilization protocols on postoperative outcomes following thoracic surgery owing to poor study quality and conflicting results [16, 17]. Additionally, the relationship between early discharge (shortened length of hospital stay) and 6MWT and postoperative walking distance, which are related to prognostic predictions, is unclear, and reports on 6MWT after surgery are few.

This study aims to clarify the relationship between walking distance within the first 24 h following lung cancer surgery and clinical outcomes. This study will help to clarify the relationship between the timing and content of postoperative walking training and overall endurance at discharge, aiding in the establishment of rehabilitation treatment after lung cancer surgery. We hypothesized that the walking distance within 24 h after surgery is longer when the 6MWT before admission and at discharge is longer, and the LOS is shorter.

## Subjects

### Study participants

This retrospective cohort study was conducted at a single center. The participant underwent respiratory surgery at a single facility between June 2020 and May 2023 and were referred to the rehabilitation department. Inclusion criteria involved patients diagnosed with lung cancer, such as small cell lung cancer or NSCLC and those who underwent a physical function assessment, including the 6MWT, by a physical therapist on the day of admission, the following day, or before discharge. Exclusion criteria included patients diagnosed with malignant pleural mesothelioma or metastatic lung cancer and those who did not undergo all physical function assessments, including the 6MWT, by a physical therapist on the day of admission, the next day, or before discharge. This Epidemiological Research Ethics Committee of our institution approved this study (approval number E2024-0172).

## Methods

### Physical function assessment

Physical function was assessed preoperatively on the day of admission or the following day and from the day before discharge to 2 days before discharge. Assessment items included grip strength on both sides (maximum value after two measurements) and 6MWT score. Grip strength was measured for 5 s in a standing position with the shoulder joints slightly externally rotated and the arms hanging down at 0° flexion and extension, without recoil. The 6MWT was performed based on the American Thoracic Society guidelines [18]. In this study, a 30 m walking course with cones was set up for participants to walk back and forth. Assessment items were the distance walked continuously, minimum percutaneous oxygen saturation during the test, percutaneous oxygen saturation at 100 m of walking, and Borg scale (lower limbs and breath) after the test.

### Other assessments

Basic data such as sex, age, height, and weight were obtained. Data on diagnosis, stage classification, preoperative chemotherapy, postoperative introduction of home oxygen therapy, and length of hospital stay were collected from medical records. Additionally, information regarding surgical site, procedure type, duration, pain management method (patient-controlled epidural analgesia or intravenous patient-controlled analgesia), postoperative complications, and comorbidities were collected from surgical records.

### Postoperative rehabilitation program

Guidelines strongly recommend mobilization within the first 24 h [19]. Early termination of bed rest/immobilization reduces pulmonary complications such as atelectasis, pneumonia, and venous thromboembolism. In our facility, mobilization was initiated by the operating physician and nurse on the morning after surgery; the urinary catheter was removed if the patient could stand and walk with an intravenous stand. Subsequently, a physical therapist visited the patient’s room to conduct walking training. Before initiating walking training, vital signs (such as blood pressure, percutaneous oxygen saturation, and respiratory status) were assessed. During this process, patients wore a chest drain and patient-controlled analgesia. No leakage was observed from the chest drain during quiet breathing; the percutaneous oxygen saturation at rest was 95% or higher; the systolic blood pressure in sitting and standing positions did not decrease by more than 30 mmHg from the supine position; and no discomfort owing to analgesia or orthostatic hypotension was observed. The attending physical therapist determined the walking distance based on the patient’s pain status and vital signs. The cumulative distance walked during the rehabilitation session was evaluated with one lap around the ward considered as 100 m. The initial walking training was conducted on the morning of the following day, regardless of whether the surgery on the previous day was performed in the morning or afternoon, as long as there were no issues regarding patient vital signs.

### Statistical analysis

Statistical analyses were performed using IBM SPSS Statistics ver. 27.0. The correlation between walking distance on the day after surgery and each assessment item was examined using Pearson’s correlation coefficient. Unpaired t-tests and chi-squared tests without correction were performed to compare basic information between participants who walked less than 100 m and those who walked 400 m or more. The level of statistical significance was set at less than 5%.

## Results

### Study participants

**Fig. 1:**
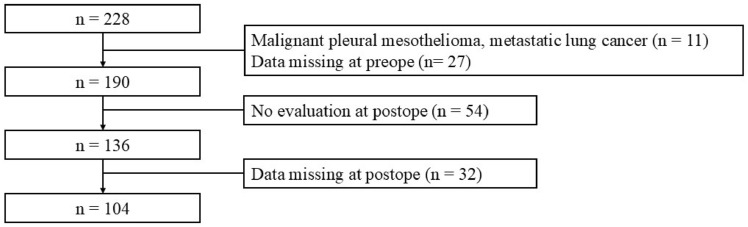
Flowchart of participants

During this period, 228 patients were admitted to the Department of Thoracic Surgery and underwent preoperative consultations in the Department of Rehabilitation. Among them, 11 had malignant pleural mesothelioma or metastatic lung tumors and 27 could not undergo preoperative physical function assessments. Postoperative reevaluation before discharge was impossible in 64 patients. After excluding 32 patients with missing data from the 136 patients assessed, 104 patients were analyzed. Figure 1 illustrates the flowchart for participant selection, and Table 1 presents basic information. All surgeries were performed using hybrid video-assisted thoracoscopic surgery. Sivelestat Sodium was administered in all cases with interstitial pneumonia complications.

**Table 1:** Characteristics of participants

Variables	All participants (*n* = 104)
Age (years)	71.7 ± 8.7
Height (cm)	160.8 ± 9.6
Weight (kg)	58.6 ± 11.6
BMI	22.5 ± 3.3
Sex (men/women)	67/37
Smoking history (Yes/no)	73/31
Stages of lung cancer (1A/1B/2A/2B/3A/3B/4A/4B)	64/21/4/3/8/1/2/1
Spirometry (normal/FEV1.0% < 70/FVC < 80/FEV1.0% < 70 and FVC < 80)	59/34/9/2
% DLco	72.8 ± 17.3
Preoperative chemotherapy (yes/no)	20/84
Frailty^a^ (yes/no)	5/99
Comorbidity	
Interstitial pneumonia	26/78
COPD/asthma	8/96
Hypertension	43/61
Diabetes mellitus	16/88
Coronary artery disease	18/86
Preoperative brain metastases	6/98
Malignant tumor, cancer	35/69
Musculoskeletal disease	10/94
Operative time (min)	138.1 ± 56.6
Operating methods	
Lobectomy	57
Segmentectomy	32
Wedge resection	15
Others	0
Laterality and lobe (right/left)	
Right upper lobe	29
Right middle lobe	9
Right lower lobe	20
Left upper lobe	25
Left lower lobe	21
PCA (PCEA/iv-PCA/none)	85/17/2
Pain at initiating walking training (numerical rating scale)	4.1 ± 3.1
Using Sivelestat sodium hydrate (yes/no)	25/79
Using home oxygen therapy (yes/no)	1/103
Postoperative drainage (day)	4.0 ± 2.4
Hospitalization (from operation to enter the hospital)	8.2 ± 3.3
Complications (yes/no)	18/86
Pulmonary fistula, prolonged air leaks	12
Atelecthasis	1
Pneumonia	2
Bowel obstruction	1
Tachycardia, atrial fibrillation	2
Walking distance at day 1 (m)	100.2 ± 75.3

Mean ± SD

*DLco* diffusion capacity of lungs for carbon monoxide, *PCA* patient-controlled analgesia

^a^A handgrip strength of less than 26 kg for men and less than 18 kg for women as well as a walking speed of less than 1.0 m/s

### Relationship between walking distance at day 1 and clinical outcome

Table 2 presents the correlation between walking distance on day 1 after surgery and clinical outcomes, as well as walking distances before and after surgery 6MWT. Significant correlations were found between walking distance on day 1 for the operative time (min), preoperative walking distance (m), and postoperative walking distance (m) (*p* < 0.05, Table 2). No significant differences were observed in terms of hospitalization (from operation to discharge).

**Table 2:** The relationship between walking distance at day 1 and clinical outcome

Variables		1	2	3	4	5	6	7	8
1	% DLco	1							
2	Postoperative drainage (day)	− 0.128	1						
3	Hospitalization (from operation to enter the hospital)	− 0.220*	0.477**	1					
4	Operative time (min)	− 0.740	0.750	0.312**	1				
5	Walk distance at preoperative (m)	0.410**	− 0.360	− 0.230*	0.680	1			
6	Walk distance at postoperative (m)	0.334**		− 0.147	− 0.380	0.690**	1		
7	Δ Walk distance (m)	− 0.660	0.430	0.560	− 0.133	− 0.326**	0.460**	1	
8	Walking distance at Day 1 (m)	0.150	0.160	− 0.147	− 0.196*	0.224*	0.397**	0.244*	1

Bold values indicate *p* < 0.05

*DLco* diffusion capacity of lungs for carbon monoxide

**Significance probability < 1%. *Significance probability < 5%

### Comparison of groups walking less than 100 m and more than 100 m on the day after surgery

Based on the mean walking distance at Day 1 (m) on 100 m for all participants, clinical outcomes were compared between groups walking less than 100 m and more than 100 m. The group that walked < 100 m had a significantly shorter walking distance in the 6MWT before and after surgery than the other groups (Table 3). Additionally, hospitalization (from operation to discharge) and days from surgery to measurement at post-operation was significantly longer, diffusion capacity of lungs for carbon monoxide (% DLco) was lower, operative time (min) was longer, and the usage rate of Sivelestat Sodium was higher in the group that walked < 100 m than those that walked > 100 m (Table 4).

**Table 3:** Comparison of groups walking less than 100 m and more than 100 m on the day after surgery

Variables		All participants (*n* = 104)	100 m < (*n* = 38)	100 m > = (*n* = 66)	*p* value
Pre-operation	Grip strength (kg)				
	Right side	27.8 ± 9.3	27.7 ± 10.6	27.8 ± 8.6	0.980
	Left side	26.7 ± 9.4	26.2 ± 10.5	26.9 ± 8.9	0.708
	6MWT				
	Walk distance (m)	466.9 ± 90.2	441.7 ± 96.7	481.4 ± 83.5	**0.030**
	Borg scale (breath)	12.3 ± 2.1	12.3 ± 2.8	12.3 ± 1.5	0.935
	Borg scale (lower leg)	11.6 ± 2.3	11.8 ± 3.0	11.5 ± 1.7	0.638
	Minimum SpO_2_	94.6 ± 2.5	94.3 ± 3.0	94.8 ± 2.3	0.300
	SpO_2_ at 100 m	94.9 ± 8.6	95.5 ± 1.9	94.5 ± 10.8	0.573
	Days from surgery to measurement	6.9 ± 3.4	8.4 ± 4.6	6.0 ± 2.0	** < 0.001**
Post-operation	Grip strength (kg)				
	Right side	26.8 ± 9.2	26.7 ± 10.4	26.9 ± 8.6	0.926
	Left side	25.4 ± 9.0	24.9 ± 10.1	25.8 ± 8.4	0.636
	6MWT				
	Walk distance (m)	383.1 ± 96.0	343.2 ± 105.9	406.1 ± 82.1	**0.001**
	Borg scale (breath)	13.6 ± 1.7	14.0 ± 1.9	13.4 ± 1.5	0.089
	Borg scale (lower leg)	11.8 ± 1.7	12.0 ± 1.6	11.7 ± 1.8	0.472
	Minimum SpO_2_	92.3 ± 3.5	91.7 ± 3.7	92.6 ± 3.3	0.182
	SpO_2_ at 100 m	94.1 ± 3.2	94.2 ± 3.0	94.1 ± 3.3	0.865
Δ walk distance in 6MWT (m)		− 83.7 ± 73.5	− 98.5 ± 76.8	− 75.3 ± 70.7	0.122
Walking distance at day 1 (m)		100.2 ± 75.3	26.9 ± 24.6	142.4 ± 60.9	** < 0.001**

Mean ± SD

Bold values indicate *p* < 0.05

*6MWT* six-minute walk test, *SpO*_*2*_ saturation of percutaneous oxygen

**Table 4:** Comparison of characteristics between walking less than 100 m and more than 100 m on the day after surgery

Variables	100 m < (*n* = 38)	100 m > = (*n* = 66)	*p* value
Age (years)	73.5 ± 9.5	70.7 ± 8.1	0.115
Height (cm)	160.7 ± 10.3	161.0 ± 9.3	0.856
Weight (kg)	59.3 ± 14.1	58.2 ± 10.1	0.638
BMI	22.7 ± 3.8	22.3 ± 3	0.630
Sex (men/women)	27/11	40/26	0.284
Smoking history (yes/no)	30/8	43/23	0.139
Stages of lung cancer (1A/1B/2A/2B/3A/3B/4A/4B)	20/5/ 3/3/6/0/1/1	44/16/1/1/ 2/1/1/0	0.060
Spirometry (normal/FEV1.0% < 70/FVC < 80/FEV1.0% < 70 and FVC < 80)	22/10/5/1	37/24/4/1	0.509
% DLco	67.5 ± 17	75.8 ± 17	**0.019**
Preoperative chemotherapy (yes/no)	7/31	13/53	0.874
Frailty^a^ (yes/no)	3/35	2/64	0.307
Comorbidity			
Interstitial pneumonia	14/ 24	12/ 54	**0.034**
COPD/asthma	5/33	3/63	0.112
Hypertension	17/21	26/40	0.594
Diabetes mellitus	7/31	9/57	0.515
Coronary artery disease	10/28	8/58	0.065
Preoperative brain metastases	2/36	4/62	0.867
Malignant tumor, cancer	13/25	22/44	0.927
Musculoskeletal disease	3/35	7/59	0.652
Operative time (min)	152.8 ± 68.5	129.7 ± 47.1	**0.001**
Operating methods			0.753
Lobectomy	22	35	
Segmentectomy	10	22	
Wedge resection	6	9	
Laterality and lobe (right/left)			0.851
Right upper lobe	10	19	
Right middle lobe	2	7	
Right lower lobe	7	13	
Left upper lobe	10	15	
Left lower lobe	9	12	
PCA (PCEA/iv-PCA/none)	28/9/1	57/8/1	0.272
Pain at initiating walking training (numerical rating scale)	4.6 ± 3.0	3.8 ± 3.1	0.112
Using Sivelestat sodium hydrate (yes/no)	14/24	11/55	**0.020**
Using home oxygen therapy (yes/no)	1/37	0/66	0.185
Postoperative drainage (day)	4.2 ± 2.8	3.8 ± 2.1	0.420
Hospitalization (from operation to enter the hospital)	9.6 ± 4.6	7.4 ± 1.9	**0.030**
Complications (yes/no)	10/28	8/58	
Pulmonary fistula, prolonged air leaks	5	7	0.695
Atelecthasis	1	0	0.185
Pneumonia	1	1	0.690
Bowel obstruction	1	0	0.185
Tachycardia, atrial fibrillation	2	0	0.057

Bold values indicate *p* < 0.05

*PCA* patient controlled analgesia, *PCEA* patient controlled epidural analgesia

^a^Handgrip strength of less than 26 kg for men and less than 18 kg for women as well as a walking speed of less than 1.0 m/s

## Discussion

In this study, walking distance within 24 h postoperatively showed a significant correlation with the preoperative and postoperative 6-min Walk Test distance (6MWD), change in 6MWD, and duration of surgery. However, no significant relationship was discovered with postoperative complications or LOS. In contrast, a comparison of groups walking less than 100 m and > 100 m the day after surgery revealed that the LOS was significantly shorter in the group walking less than 100 m and the usage rate of Sivelestat Sodium was higher. This study represents a novel investigation comparing walking distance immediately after surgery with clinical outcomes and is the first to enable quantitative evaluation of early postoperative mobilization.

A significant correlation was observed between walking 100 m on the first post and the preoperative 6MWD. This finding suggests that patients with lung cancer with sufficient exercise tolerance before surgery are more likely to achieve early mobilization. Perioperative rehabilitation can prevent postoperative complications [20]. For example, maximum oxygen uptake (VO_2_ max) of > 15 mL/kg/min before surgery is associated with lower mortality rates [21]. Positive correlation between the 6-min Walk Test distance and *V*O_2_ max in patients with chronic obstructive pulmonary disease [22], indicating that extending the 6MWD before surgery may lead to an increase in VO_2_ max and facilitate smoother early postoperative mobilization.

Previous studies on early mobilization have included mobilization within 24 h in the ERAS [10]; however, no reports have used specific distances as milestones. In studies defining early walking as occurring within 24 h, the early walking group had significantly fewer postoperative complications [11]. In our study, the average walking distance on the day after surgery exceeded 100 m; however, no significant difference in postoperative complications was observed between those who walked 100 m and those who did not (*p* = 0.065). Therefore, achieving a walking distance of 100 m within 24 h postoperatively may be recommended as part of ERAS. However, a correlation was observed between the 100 m walking distance and duration of surgery. Patients unable to walk 100 m within 24 h postoperatively had a significantly higher use of Sivelestat Sodium. At our institution, Sivelestat Sodium is administered in cases with interstitial pneumonia to prevent the deterioration of respiratory function. Therefore, the inability to walk within 24 h after surgery is considered to be due to preoperative interstitial pneumonia and the resulting decline in overall endurance. In addition, the group that started walking after 24 h after surgery had more complications and longer drainage periods. Therefore, walking 100 m within 24 h after surgery should not be treated as a goal, but rather as a predictive index based on the influence of preoperative factors and hospital stay duration.

No studies have reported 6MWD at discharge after lung cancer surgery. In patients with concurrent chronic obstructive pulmonary disease and lung cancer, the 6MWD decreases by approximately 43 m at 1 month after surgery (from 412.0 ± 27.3 preoperatively to 369.0 ± 33.8 m at 1 month) [23]. They reported minimal clinically important difference in 6MWD for patients with lung cancer ranges from 22.0 to 42.0 m [24]. In our study, the average decrease was 83 m, with a significant correlation between the change in 6MWD and pre- and postoperative distances. This finding indicates that early decline in the 6MWD following lung cancer surgery are related to preoperative physical function. However, no significant difference in the change was observed between groups that walked 100 m within 24 h and those that did not, with higher preoperative and postoperative 6MWD in the 100-m walking group. This finding suggests that a decline in physical function can occur even with extended early walking distance.

In this study, the groups were divided based on an average walking distance of 100 m. Few studies have reported average walking distances within 24 h after lung cancer surgery, leaving room for debate regarding the adoption of 100 m as the ERAS criterion. A significant difference is observed in the 5-year survival rates when the preoperative 6-MWD is less than 400 m in patients with NSCLC compared with those with a walking distance of 400 m or more [25]. Furthermore, a linear relationship has been observed between diffusing DLco and oxygen consumption (VO_2_) up to approximately 70% of *V*O_2_max in healthy participants performing incremental exercises [26]. In this study, participants unable to walk 100 m on the first postoperative day also had low DLco values and interstitial pneumonia. Based on evidence that rehabilitation improves exercise tolerance in interstitial lung disease [27], treatment for interstitial pneumonia and maximizing oxygen intake through rehabilitation before surgery may be related to prognosis after lung cancer resection. In our study, the group that walked within 24 h postoperatively had an average walking distance of 400 m, suggesting that walking 100 m could serve as an outcome indicator following lung cancer surgery. In contrast, another report indicated that a group capable of walking for more than 5 min within 1 h after extubation experienced significantly reduced LOS [27], suggesting that initiating mobilization earlier and extending walking distances could further improve clinical outcomes after lung cancer surgery. However, differences in clinical outcomes are recognized only with or without perioperative interventions such as ERAS protocols. This study also observed significant differences in preoperative and postoperative 6-MWD and the 24-h postoperatively. Patients who could not walk 100 m postoperatively exhibited lower pulmonary diffusion capacity, suggesting that preventing postoperative complications following lung cancer surgery is influenced by preoperative exercise tolerance. This finding aligns with that of a meta-analysis showing that combining preoperative respiratory rehabilitation with aerobic exercise can reduce LOS [28]. The clinical significance of this study is that walking 100 m on the first postoperative day is influenced by preoperative physical function (pulmonary diffusion capacity and overall endurance), and insufficient walking ability on the first postoperative day indicates that adequate physical function may not be regained by the time of discharge. This study suggests that preoperative rehabilitation to improve physical function, particularly endurance [29], is crucial for promoting early discharge, and that early postoperative walking training is necessary to prevent declines in physical function after discharge.

The limitations of this study include its retrospective cohort design, which did not clarify the causal relationship between walking distance, LOS, and complications. Additionally, this single-center study included few participants with a preoperative 6MWD below 400 m and it was not an interventional study to determine whether the rehabilitation program reduced LOS or prevented complications. Furthermore, the walking distance on the day after surgery was left to each physical therapist without setting clear criteria; thus, variations in therapist proficiency may have affected the results. Moreover, cases complicated by interstitial pneumonia accounted for a relatively high proportion (25%), exceeding the level reported in our department’s previous study (15%) [30] and in reports from other institutions [31]. Additionally, interstitial pneumonia was more frequently observed in cases where patients were unable to walk 100 m on the day following surgery. These findings suggest that the presence of interstitial pneumonia may influence clinical outcomes based on walking ability, which remains a limitation that cannot be ruled out. In addition, differences in LOS between the two groups resulted in differences in the timing of the postoperative 6MWT. Since physical function improves daily after lung cancer surgery, a difference of more than 1 day may affect physical function. Therefore, the 6MWT walking distance of the group able to walk 100 m on the day after surgery, which had shorter hospital stays, may have been underestimated. Further, differences in hospital stay length and preoperative conditions (such as low %DLco and interstitial pneumonia) could serve as confounding factors, representing a limitation of this retrospective cohort study.

## Conclusion

This study examined the relationship between early postoperative walking distance and clinical outcomes in patients who underwent lung cancer surgery. Our results suggest that early postoperative mobilization, specifically walking 100 m within the first 24 h, is an important indicator of physical function and recovery. Patients with better preoperative endurance are more likely to achieve early mobilization, which may lead to improved overall outcomes. Early walking did not reduce postoperative complications or LOS; however, it served as a useful marker for assessing the recovery potential. Preoperative physical fitness plays a key role in postoperative recovery, and rehabilitation programs should focus on enhancing endurance before surgery. Further studies are needed to refine early mobilization protocols and fully understand their impact on clinical outcomes after lung cancer surgery.

## Data Availability

The datasets used and analyzed during the current study are available from the corresponding author upon reasonable request.

## References

[1] Cancer statistics, Cancer information service, National Cancer Center Japan [Internet]. https://ganjoho.jp/reg_stat/statistics/stat/cancer/12_lung.html. Accessed 4 Oct 2023.

[2] Liang Z, Li X, Li X. Survival analysis of surgical versus nonsurgical treatment in stage I to III small cell lung cancer in the last 20 years: a systematic review and meta-analysis. Thorac Cancer. 2023;14:2525–35. 37567777 10.1111/1759-7714.15062 PMC10481146

[3] American College of Sports Medicine, Liguori G, Feito Y, Fountaine CJ, Roy B, editors. ACSM’s guidelines for exercise testing and prescription. Eleventh edition. Philadelphia Baltimore New York London$PBuenod Aires Hong Kong Sydney Tokyo: Wolters Kluwer; 2022.

[4] van der Leeden M, Balland C, Geleijn E, Huijsmans RJ, Dekker J, Paul MA, et al. In-hospital mobilization, physical fitness, and physical functioning after lung cancer surgery. Ann Thorac Surg. 2019;107:1639–46. 30690020 10.1016/j.athoracsur.2018.12.045

[5] Marjanski T, Wnuk D, Dziedzic R, Ostrowski M, Sawicka W, Rzyman W. 500 meters is a result of 6-minute walk test which differentiates patients with high and low risk of postoperative complications after lobectomy—a validation study. J Clin Med. 2021;10:1686. 33919996 10.3390/jcm10081686 PMC8070994

[6] Oikawa M, Hanada M, Nagura H, Tsuchiya T, Matsumoto K, Miyazaki T, et al. Factors influencing functional exercise capacity after lung resection for non-small cell lung cancer. Integr Cancer Ther. 2020;19:1534735420923389. 32493079 10.1177/1534735420923389 PMC7273541

[7] Ni H-J, Pudasaini B, Yuan X-T, Li H-F, Shi L, Yuan P. Exercise training for patients pre- and postsurgically treated for non-small cell lung cancer: a systematic review and meta-analysis. Integr Cancer Ther. 2017;16:63–73. 27151583 10.1177/1534735416645180 PMC5736064

[8] Kehlet H, Wilmore DW. Multimodal strategies to improve surgical outcome. Am J Surg. 2002;183:630–41. 12095591 10.1016/s0002-9610(02)00866-8

[9] Smart NJ, White P, Allison AS, Ockrim JB, Kennedy RH, Francis NK. Deviation and failure of enhanced recovery after surgery following laparoscopic colorectal surgery: early prediction model. Colorectal Dis. 2012;14:e727-734. 22594524 10.1111/j.1463-1318.2012.03096.x

[10] Rogers LJ, Bleetman D, Messenger DE, Joshi NA, Wood L, Rasburn NJ, et al. The impact of enhanced recovery after surgery (ERAS) protocol compliance on morbidity from resection for primary lung cancer. J Thorac Cardiovasc Surg. 2018;155:1843–52. 29352586 10.1016/j.jtcvs.2017.10.151

[11] Ding X, Zhang H, Liu H. Early ambulation and postoperative recovery of patients with lung cancer under thoracoscopic surgery-an observational study. J Cardiothorac Surg. 2023;18:136. 37041603 10.1186/s13019-023-02263-9 PMC10091666

[12] Nakada T, Shirai S, Oya Y, Takahashi Y, Sakakura N, Ohtsuka T, et al. Four hours postoperative mobilization is feasible after thoracoscopic anatomical pulmonary resection. World J Surg. 2021;45:631–7. 33098011 10.1007/s00268-020-05836-0

[13] Unaldi HE. Effects of mobilization within the first 4 h following anatomical lung resection with thoracotomy. Updates Surg. 2023;75:2027–31. 37524991 10.1007/s13304-023-01617-1

[14] Wade-Mcbane K, King A, Urch C, Jeyasingh-Jacob J, Milne A, Boutillier CL. Prehabilitation in the lung cancer pathway: a scoping review. BMC Cancer. 2023;23:747. 37568130 10.1186/s12885-023-11254-x PMC10416419

[15] Zhou W, Woo S, Larson JL. Effects of perioperative exercise interventions on lung cancer patients: an overview of systematic reviews. J Clin Nurs. 2020;29:4482–504. 32979874 10.1111/jocn.15511

[16] Castelino T, Fiore JF, Niculiseanu P, Landry T, Augustin B, Feldman LS. The effect of early mobilization protocols on postoperative outcomes following abdominal and thoracic surgery: a systematic review. Surgery. 2016;159:991–1003. 26804821 10.1016/j.surg.2015.11.029

[17] Mainini C, Rebelo PF, Bardelli R, Kopliku B, Tenconi S, Costi S, et al. Perioperative physical exercise interventions for patients undergoing lung cancer surgery: what is the evidence? SAGE Open Med. 2016;4:2050312116673855. 27803808 10.1177/2050312116673855 PMC5077072

[18] ATS Committee on Proficiency Standards for Clinical Pulmonary Function Laboratories. ATS statement: guidelines for the six-minute walk test. Am J Respir Crit Care Med. 2002;166:111–7. 12091180 10.1164/ajrccm.166.1.at1102

[19] Batchelor TJP, Rasburn NJ, Abdelnour-Berchtold E, Brunelli A, Cerfolio RJ, Gonzalez M, et al. Guidelines for enhanced recovery after lung surgery: recommendations of the enhanced recovery after surgery (ERAS^®^) Society and the European Society of Thoracic Surgeons (ESTS). Eur J Cardiothorac Surg. 2019;55:91–115. 30304509 10.1093/ejcts/ezy301

[20] Gravier F-E, Smondack P, Prieur G, Medrinal C, Combret Y, Muir J-F, et al. Effects of exercise training in people with non-small cell lung cancer before lung resection: a systematic review and meta-analysis. Thorax. 2022;77:486–96. 34429375 10.1136/thoraxjnl-2021-217242

[21] Arbee-Kalidas N, Moutlana HJ, Moodley Y, Kebalepile MM, Motshabi CP. The association between cardiopulmonary exercise testing and postoperative outcomes in patients with lung cancer undergoing lung resection surgery: a systematic review and meta-analysis. PLoS One. 2023;18: e0295430. 38060569 10.1371/journal.pone.0295430 PMC10703215

[22] Chuang ML, Lin IF, Wasserman K. The body weight-walking distance product as related to lung function, anaerobic threshold and peak *V*O_2_ in COPD patients. Respir Med. 2001;95:618–26. 11453321 10.1053/rmed.2001.1115

[23] Ogura R, Iribe S, Suzuki T, Kuroda H, Sugisawa T, Okuni I, et al. Persistence of the postoperative change in the six-minute walking distance of lung cancer patients with chronic obstructive pulmonary disease. Prog Rehabil Med. 2021;6:20210022. 34013089 10.2490/prm.20210022 PMC8103386

[24] Granger CL, Holland AE, Gordon IR, Denehy L. Minimal important difference of the 6-minute walk distance in lung cancer. Chron Respir Dis. 2015;12:146–54. 25749346 10.1177/1479972315575715

[25] Hamada K, Irie M, Fujino Y, Hyodo M, Hanagiri T. Prognostic value of preoperative exercise capacity in patients undergoing thoracoscopic lobectomy for non-small cell lung cancer. Lung Cancer. 2019;128:47–52. 30642452 10.1016/j.lungcan.2018.12.013

[26] Rampulla C, Marconi C, Beulcke G, Amaducci S. Correlations between lung-transfer factor, ventilation, and cardiac output during exercise. Respiration. 1976;33:405–15. 1005935 10.1159/000193758

[27] Dowman L, Hill CJ, Holland AE. Pulmonary rehabilitation for interstitial lung disease. Cochrane Database Syst Rev. 2014. 10.1002/14651858.CD006322.pub3. 25284270 10.1002/14651858.CD006322.pub3

[28] Zhou C, Luo Y, Pan X, Xia F, Li M, Li W. Early enhanced recovery after lung surgery: early ambulation 1 hour after extubation. Ann Palliat Med. 2021;10:9732–41. 34628899 10.21037/apm-21-2102

[29] Pu CY, Batarseh H, Zafron ML, Mador MJ, Yendamuri S, Ray AD. Effects of preoperative breathing exercise on postoperative outcomes for patients with lung cancer undergoing curative intent lung resection: a meta-analysis. Arch Phys Med Rehabil. 2021;102:2416-2427 (**e4**). 33930327 10.1016/j.apmr.2021.03.028 PMC9353730

[30] Fujiwara M, Mimae T, Tsutani Y, Miyata Y, Okada M. Complications and survival after lung cancer resection in interstitial lung disease. Ann Thorac Surg. 2023;115:701–8. 35863399 10.1016/j.athoracsur.2022.05.069

[31] Kakiuchi S, Hanibuchi M, Tezuka T, Saijo A, Otsuka K, Sakaguchi S, et al. Analysis of acute exacerbation of interstitial lung disease associated with chemotherapy in patients with lung cancer: a feasibility of S-1. Respir Investig. 2017;55:145–52. 28274530 10.1016/j.resinv.2016.10.008

